# Effect of surface morphology on optical properties of two multilayer structures CuO/ZnO/SiC and Al_2_O_3_/ZnO/SiC

**DOI:** 10.1038/s41598-023-48998-2

**Published:** 2023-12-27

**Authors:** H. Abd El-Fattah

**Affiliations:** Department of Manufacturing Engineering and Production Technology, Modern Academy for Engineering and Technology, Cairo, Egypt

**Keywords:** Engineering, Materials science, Nanoscience and technology, Physics

## Abstract

Zinc oxide (ZnO) and Silicon carbide (SiC) thin films demonstrate unique properties such as high electron mobility, thermal stability, good chemical resistance, and low cost made them good candidates for optical applications. Moreover, semiconductors absorb short wavelengths of light due to the presence of a band gap. This work’s purpose is to study the effect of deposited ZnO and SiC thin films by physical vapor deposition (PVD) above two different oxides and substrates. Copper (Cu) with copper oxide (CuO) and aluminum (Al) with aluminum oxide (Al_2_O_3_) were the used substrates and oxides. After deposition of thin films, two different multilayer structures were resulted, which are CuO/ZnO/SiC and Al_2_O_3_/ZnO/SiC. Microstructure and morphology were investigated by scanning electron microscope (SEM) and atomic force microscope (AFM). Structure and phases identification were examined by X-ray diffraction (XRD). Optical properties (absorbance and emittance) before and after depositions of thin films were measured by spectrophotometer and Fourier transform infrared spectroscopy (FTIR). The results showed that the CuO/ZnO/SiC structure (85%) had higher absorbance than Al_2_O_3_/ZnO/SiC structure, however Al_2_O_3_/ZnO/SiC showed higher selectivity (absorbance/emittance (α/ε)) of about 0.65/0.15, compared to 0.85/0.5 for CuO/ZnO/SiC multilayer structure. The effect of surface topography and roughness on the efficiency of each multilayer structure has been studied.

## Introduction

Concentrating solar power (CSP) manufacturing has gained a great attention in the recent years due to a highly increasing need of clean and renewable energy. Solar energy is an abundant resource distributed and radiated in all regions of the world. A lot of research intended to enhance the mirrors, focal point, angles of parabolic receiver, and selective absorber of CSP. This work is focused on the selective absorber which is coated on the receiver tube to capture the solar radiation in ultraviolet (UV), visible light and near infra-red (NIR) regions. The wavelength range needed to capture is from 200 to 2500 nm. The working temperature of CSP is about (400–500) °C. To have a highly efficient solar selective absorber (SSA), it should have higher absorbance than 90% and lower emittance than 10%^1^.

There are many types of selective absorbers such as, Intrinsic, metal dielectric, and multilayer structure. Multilayer selective absorber is an interesting type to research and investigate^1^. Many reflectance passes in multilayer structure are the reason of selectivity. Multi-layer structure consisted of the infra-red (IR) layer (metallic) then the core layer (oxide or semiconductor) which consisted of more than one layer then an AR layer on the top (oxides or carbides)^2^. Many techniques are used to deposit selective coatings such as PVD direct current (DC) or radio frequency (RF), chemical vapor de-position (PECVD), painting technique and electrodeposition and chemical sol–gel. In this work, PVD was used due to depositing a highly adhesive and homogenous thin film^3^. Structure of selective absorber is dependent on the core layer which should have high absorbance and an AR layer above it made of oxides or carbides to prevent emittance of the captured light. AR layer should have low thermal conductivity and corrosion-resistant properties to provide protection and decrease the degradation^2^.

ZnO became a considerable and efficient material in solar cells third generation, due to having many advantages such as good growth control, large energy bandgap, low cost, and high electron mobility^4^. ZnO has a band gap of about 3.37 eV at room temperature with direct electron transitions and high transparency in visible light region^5^. Amakali et al. studied the deposition of ZnO by two different methods (molecular precursor and sol–gel). They investigated the structure and optical properties of ZnO and found that the thin film fabricated by molecular method was more transparent than the sol–gel one^6^. Wang et al. deposited ZnO and compared its emission with CuO/ZnO composite. The pure ZnO has recorded strong UV emission compared to CuO/ZnO composite^7^. Ismail et al. investigated the effect of RF power on the optical and structural properties of deposited ZnO thin film. They observed that as RF power increased the UV emission peak was revealed to a blue shift^8^. Sharmila et al. deposited ZnO by RF sputtering technique followed by annealing of thin film at different temperatures (100, 200, 300 °C) to investigate the stability and effect of high temperatures on optical properties of ZnO thin films. They observed that annealed thin film had the highest optical response and good results in UV range^9^.

SiC thin films have unique physical and chemical characteristics, such as high thermal stability, good chemical resistance, distinctive electronic properties, low dimensionality, and good optical properties^10^. In addition, SiC as a semi-conductor with its wide band gap can be used in new advanced applications to develop efficient UV photonic^11^. Tavsanoglu et al. produced amorphous SiC thin film on different substrates by reactive medium sputtering (CH_4_ gas was used). The flow rate of CH_4_ gas was changed to study the effect of it on the optical properties of deposited SiC thin film. They noticed that the optical and electrical characteristics of SiC thin film can be fitted by changing Si and C concentrations in thin film^12^. SiC can be deposited by different fabrication methods for example, pulsed laser deposition, direct ion deposition, and reactive DC or RF magnetron sputtering. In this work SiC was deposited directly from the target in RF magnetron sputtering without any reactive medium.

Many researches studied the deposition of antireflection layers such as ZnS and MgF_2_ thin films by PVD technique or vacuum thermal evaporation. The deposition angles in PVD technique were varied which were reflected on the crystallinity of thin film. They found that the crystallinity of the deposited thin films decreased as the deposition angle increased^13^. Also, the incident vapor flow angles were varied in vacuum thermal evaporation deposition technique. Thin film crystallinity found to be very sensitive to the growth angle^14,15^.

Multilayer selective absorbers were proposed, and many designs have been applied with different thin film materials^16–21^. For example, Tibaijuka et al.^19^ developed a multilayer stack of Al_x_O_y_/Cr/Al_x_O_y_ with an absorbance and thermal emittance of about 0.91 and 0.12 respectively at 373 K.

CuO and Al_2_O_3_ were formed naturally above copper and aluminium when they were exposed to air. CuO and Al_2_O_3_ can be used as selective coatings and they have promising properties for thermal solar applications, however CuO has stability at temperature up to 400 °C^22,23^. The optical properties (absorbance and emittance) of mentioned oxides were measured and found to be interesting. Absorbance of CuO with brown colour and Al_2_O_3_ was recorded about 90% and 78%, respectively with continuous pattern along UV, visible light, and short IR range (the whole useful range). Multilayer selective absorbers achieve higher absorbance due to the graded refractive index of deposited thin films. The novelty of this work is the deposition of ZnO thin film as an absorber layer with CuO or Al_2_O_3_ to increase absorbance by increasing paths of light, then deposition of SiC as AR layer above CuO and Al_2_O_3_ oxides to test its effect on the optical properties of them. The main role of AR layer as mentioned before is to prevent the emittance of captured light. Optical properties, structure, and topography of the surface were measured and studied before and after deposition of ZnO and SiC thin films. ZnO and SiC thin films were chosen due to their interesting characteristics of both. The performance of AR layer was great with its impressive thermal, chemical and electronic properties of SiC which make it a good candidate as AR layer.

## Materials and methods

### Thin films preparation

PROTOFEEX sputtering 1600- Magnetron 6 (USA) sputtering PVD was used in deposition of ZnO and SiC thin films. The 99.9% copper (Cu) and 99.9% aluminium (Al) substrates dimensions were 4 × 4 cm^2^ with 2 mm thickness. Chamber of deposition had bias voltage and velocity about 150 V and 10 rpm respectively. Pure ZnO and pure SiC targets (99.999%) were used in deposition. The chamber was initially down to pressure 10^−5^ bar, 100-Watt RF Power was used to sputter ZnO thin film. Time of sputtering was 2 h. While DC Power 350 V was used in SiC thin film deposition for 1 h. Deposition pressure was 10^−3^ for SiC and ZnO thin films with argon gas (Ar) flow rate 30 sccm.

**Figure 1:**
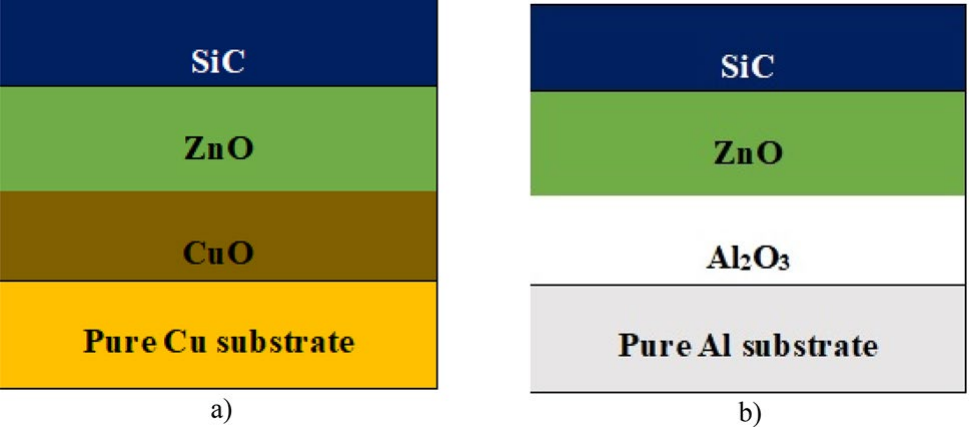
SSA multi-layers design.

Figure 1 shows the schematic drawing of the (SSA) multi-layers design deposited on pure Cu and Al substrates (Cu/CuO/ZnO/SiC–Al/Al_2_O_3_/ZnO/SiC).

### Thin films characterization

#### Surface morphology and phase identification

Scanning electron microscope (SEM) (Thermo Fisher Scientific Electron Microscopy) was used in investigating of surface and cross-section images. Surface morphology and topography were investigated by Atomic force microscope (AFM) 5600LS AFM. 3D AFM images were obtained for morphology. The surface roughness and thickness of thin films were measured.

X-Ray diffraction (XRD) was used in phase identification. Bruker model has scanning range 10 ≤ 2θ ≤ 100° intervals, step size 0.1 deg and a Cu target operating at 40 kV and 30 mA.

#### Optical properties

The absorbance and emittance were measured by using spectrophotometer and FTIR for respectively. FTIR is NICOLET 6700 model. Spectrophotometer model is Shimadzu UV-3600. Reflectance was measured in the whole range of light wavelength (0.2–25 µm) by using the two mentioned equipment. To have absorbance within (0.2–2.5 µm) range, and emittance within (2.5–25 µm) range Kirchhoff’s laws of opaque materials were applied^24^. All measurements were occurred at R.T.

## Results and discussion

### Surface microstructure, topography, and roughness

**Figure 2:**
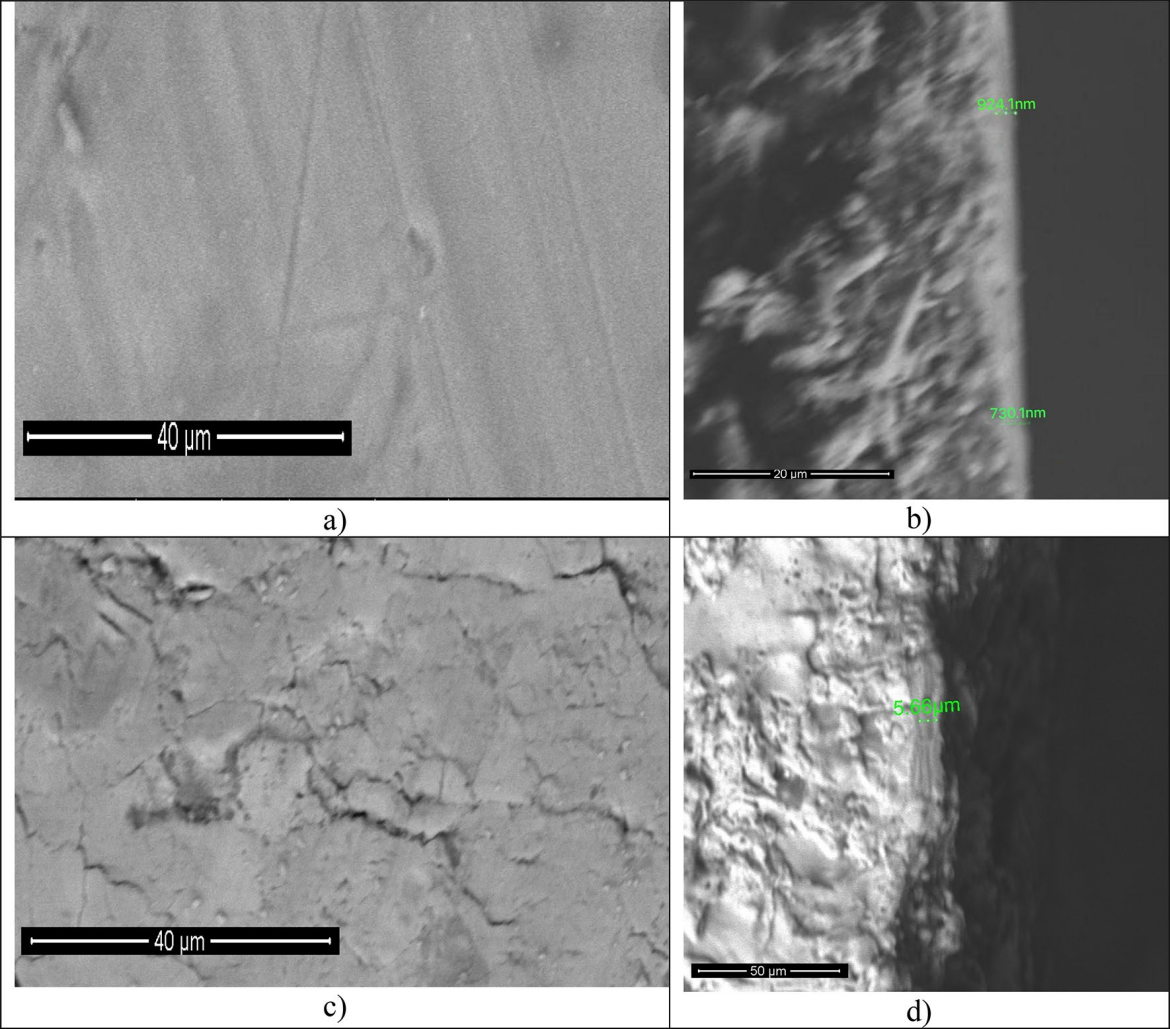
(**a**) and (**c**) SEM images of Al_2_O_3_ and CuO before deposition respectively. (**b**) and (**d**) images are a vertical cross-section of Al_2_O_3_ and CuO thicknesses respectively.

Figure 2 shows the SEM images of CuO and Al_2_O_3_ before deposition of ZnO and SiC thin films. Figure 2a presents the microstructure of Al_2_O_3_. There is an amorphous microstructure without definite grains or grain boundaries. Only the scratches that had occurred during cutting of Al substrate could be recognized^25^. Figure 2b shows a vertical cross section to substrate and Al_2_O_3_ formed above it by SEM to measure the thickness of oxide and to ensure of its presence. Two readings were recorded in two different positions 924.1 nm and 730.1 nm with average thickness of about 827.1 nm. The Cu substrate used in this work had a dark brown thin layer above it related to CuO thin film formed naturally. In Fig. 2c CuO natural thin film appeares with some scratches and cracks in it. CuO can be noticed by visual inspection, but the thickness should be measured to know the accurate dimensions of it. Figure 2d is a vertical cross section to measure the CuO thickness. It was about 5.66 µm.

Surface topography of any thin film was studied by AFM due its strong and good profilometry technicality. A lot of information about the surface can be obtained by using this technique. Roughness, grain size, and structure max., and min. heights of thin film surface can be studied through morphology images^26^.

**Figure 3:**
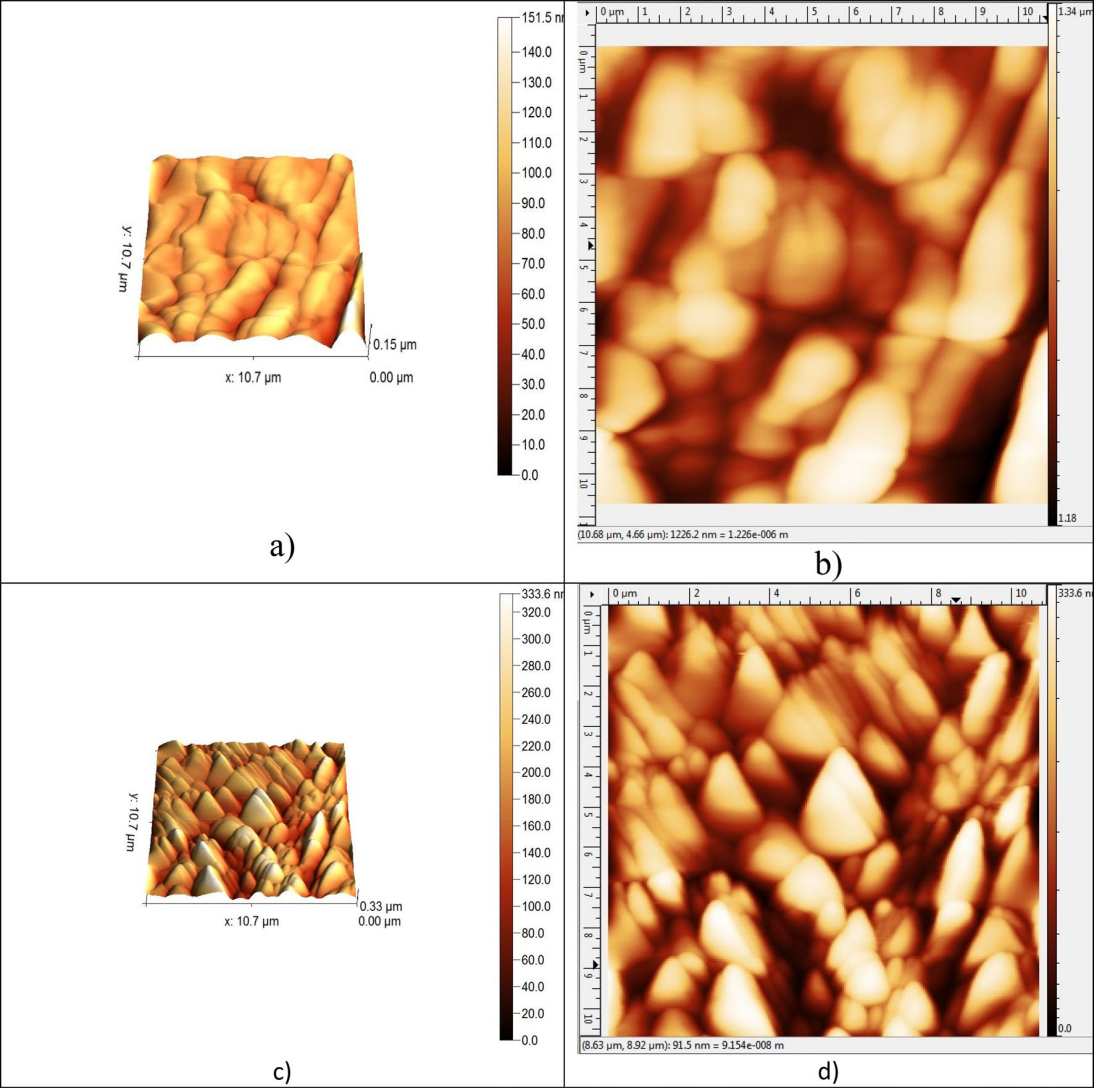
AFM images 3D and 2D profiles of SiC thin film (**a**) and (**b**) above Al_2_O_3_ thin film and (**c**) and (**d**) above CuO thin film.

The relation between evaluating structure of deposited thin film by RF sputtering and deposition conditions had been studied by structural zone models (SZM)^27^. SZM divides conditions into three zones, which are Zone I, Zone T and II. The parameters of deposition such as pressure, chamber temperature, gas flow rate, etc., which determine the three zones. According to SZM models the deposition of SiC thin film was in Zone I. At this zone, thin film microstructure is porous, may contain fine fibres, or amorphous textured, a small and more equiaxed grains may be formed^27^. The AFM images shown at Fig. 3 confirmed this assumption. XRD will be discussed later to further confirm this.

As shown in Fig. 3 the SiC on the surface in the top of multi-layer has two different morphologies according to the substrate it had been deposited on. Thin films were grown by charged particles add- atoms. Different structures and morphologies of deposited layers occurred due to the differences in rates of transferred charges caused by using different substrates^28^. SiC and ZnO thin films are deposited at the same conditions but on different substrates.

Figures 3a,b show deposited thin films above Al_2_O_3_, where homogenous wavy structure and valleys shown in Fig. 3a and clusters of particles shown in Fig. 3b. Figures 3c,d show SiC thin film on the top in which the structure of CuO nano sphere morphology is clearly found, and Zno and SiC were deposited inside it. Nano sphere clusters structure of CuO was found^29^.

Table 1 shows root mean square (Rrms), roughness average (Ra), skewness (Rsk) and kurtosis (Rku) of SiC thin film above Al_2_O_3_ and CuO. Also, average max. hight of profile (R_z_) and waviness average (W_a_) were measured.

**Table 1:** comparison of AFM data at two cases.

Data of ZnO and SiC above two oxides	Above Al_2_O_3_	Above CuO
R_rms_	1.64144 nm	9.63475 nm
R_a_	1.20430 nm	6.96701 nm
R_SK_	− 0.985212	− 0.321709
R_Ku_	7.29096	5.01900
R_z_	8.08592 nm	47.8028 nm
W_a_	17.4693 nm	40.7505 nm

As shown in Table 1 there are noticable differences in measurements of the same thin film deposited on two different oxides. These differences would reflect on optical properties and its values as will be discussed later. Skewness and kurtosis values indicated the surface features symmetry, controlled by peaks, and bumpy^30^. As shown in Table 1 the values of max. height profile and waviness average is higher in case of deposition above CuO than Al_2_O_3_. Wavy structure with clusters can be observed at two cases but it is clearer at Fig. 3c.

ZnO thin film was deposited in two cases at 100 W, but the wavy structure appeared clearer at 3D AFM Fig. 3c in which the surface is spiny and influenced by valleys. This observation matches with skewness and kurtosis values shown in Table 1. 3D AFM image (Fig. 3c) shows spines, spheres, and valleys in thin film deposited above CuO. However, the deposition conditions were the same in both cases, but the topography is not the same. This was due to the nature of CuO and Al_2_O_3_. Although, the small value of deposition watt (100 W) was the reason of surface homogeneity with formed spines and valleys^26^.

### Phase identification

**Figure 4:**
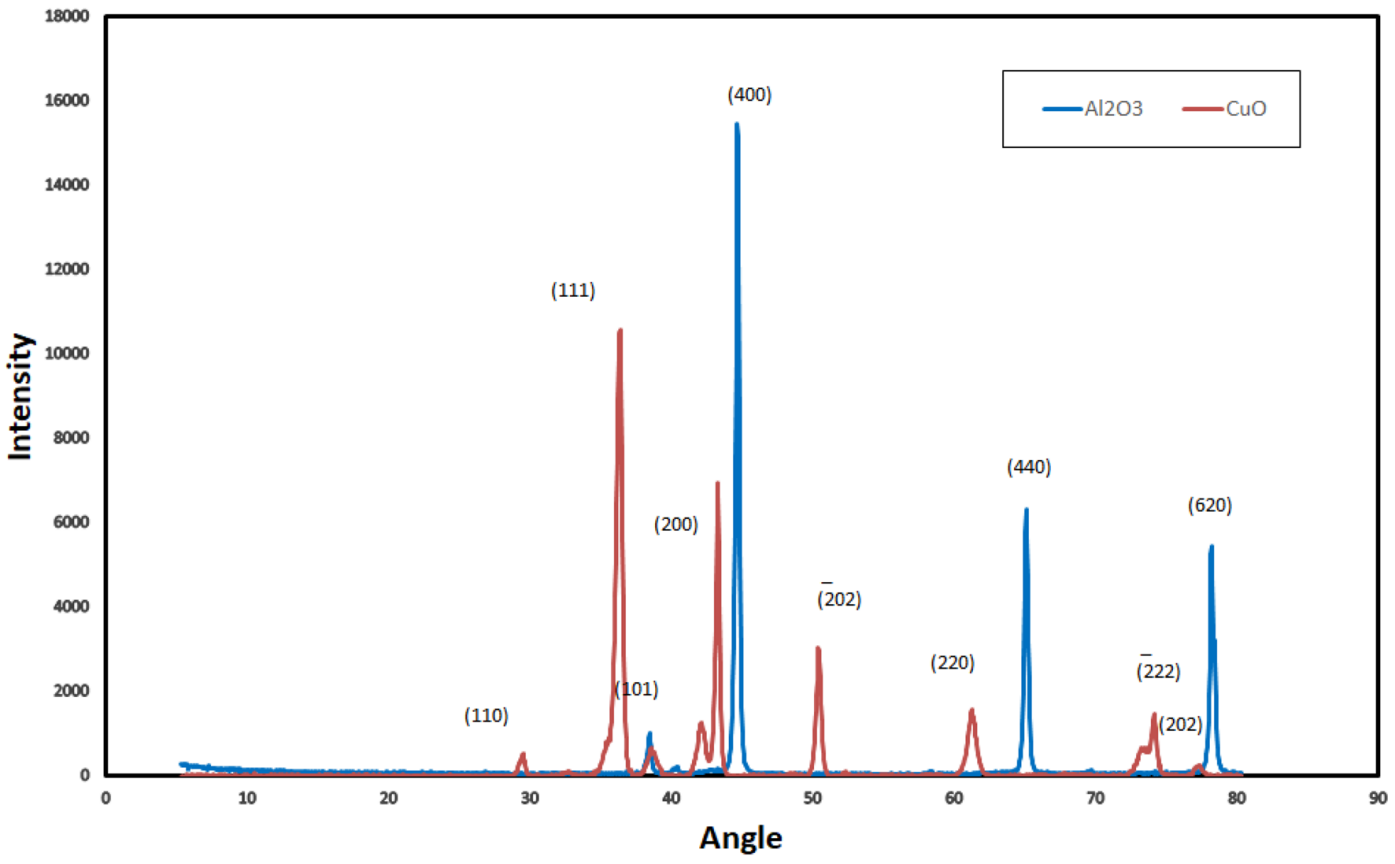
XRD of ZnO and SiC thin film above Al_2_O_3_ and CuO.

**Figure 5:**
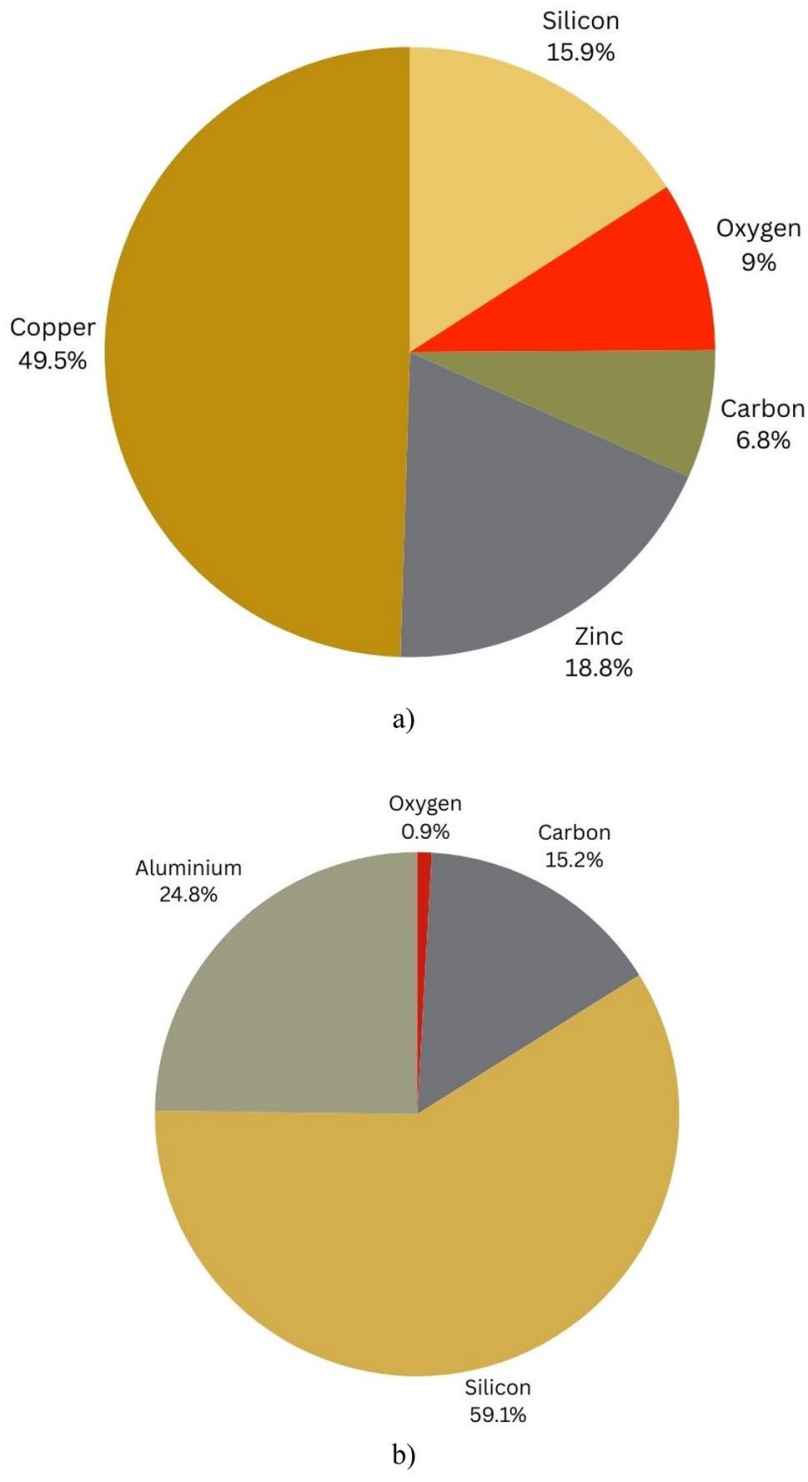
XRD elements concentration (**a**) ZnO and SiC thin film above CuO. (**b**) ZnO and SiC thin film above Al_2_O_3_.

Figure 4 shows the XRD of SiC and ZnO thin films in two cases above Al_2_O_3_ and CuO. XRD is used to determine the structure, phases, and crystallinity of materials. Table 2 highlights the major diffraction peaks angles and related phases planes. Figure 5 illustrates the XRD elements concentration. It shows the presence of Al_2_O_3_, CuO, SiC, and ZnO thin films. The obtained values are compatible with Crystallography Open Database (COD) numbers. SiC formed above CuO had hexagonal structure with lattice parameters a = 0.307 nm and c = 4.775 nm (COD 1,538,515) and above Al_2_O_3_ had two structures, hexagonal (lattice parameters a = 0.3079 nm and c = 2.518 nm (COD 2,310,851)) and cubic structure (lattice parameter a = 0.4523 nm (COD 1,536,528)). The difference that occurred in SiC structures is due to the growth mode which was strongly dependent on the surface stoichiometry^38^. The diffraction pattern angles (Fig. 4 and Table 2) at 42&43, 63 showed the formation of ß-3C SiC with the crystal planes at (200) and (220) respectively^34^. ZnO formed above CuO had cubic structure with lattice parameter a = 0.428 nm (COD 1,534,836). XRD diffraction peaks in planes (101), (202) belonged to ZnO structure^26^. A strong and sharp peak appeared at 36° angle with (111) plane which indicated the crystallinity of formed CuO^30^. The found CuO had two structures monoclinic with lattice parameters a = 0.4689 nm, b = 0.3427 nm, and c = 0.513 nm (COD 9,016,057) and cubic structure with lattice parameter a = 0.4269 nm (COD 9,005,769). Al_2_O_3_ was found with sharp and strong diffraction peaks in planes (400), (440), (620) presented the crystallinity of it^35^. It had cubic structure with lattice parameter a = 0.4049 nm (COD 4313210). As well as the lattice constant, XRD patterns give information of the average crystallite size (D). This quantity was obtained with the Debye–Scherrer’s formula^39^ Eq. (1).

1 $$D = \frac{K\lambda }{{\beta cos\theta }}$$

where k is the shape factor (0.9), λ is the wavelength of the Cu Kα, β is the full width at half maximum (FWHM) of the most intense peak of the XRD spectrum and θ is the Bragg angle. The crystalline size of CuO was about 1.534 nm and Al_2_O_3_ was about 1.25 nm, obtained from (111) and (400) width XRD peaks respectively.

**Table 2:** XRD peak angles and related phases.

Compound	Peak angle	Phase plane	Ref.
ZnO	37.5, 78	(101), (202)	^31,32^
SiC	42 & 43, 63	(200), (220)	^33,34^
Al_2_O_3_	45, 65, 79	(400), (440), (620)	^35^
CuO	30, 36, 50, 75	$(110),(111),(\overline{2}02),(2\overline{2}2)$	^36,37^

### Optical properties

**Figure 6:**
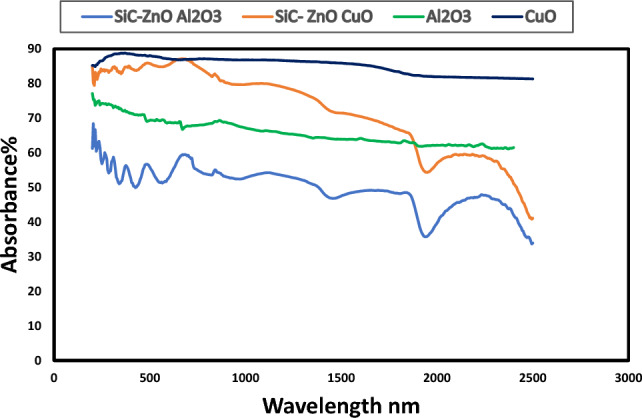
Absorbance of deposited SiC and ZnO above Al_2_O_3_ and CuO and two oxides Al_2_O_3_ and CuO.

**Figure 7:**
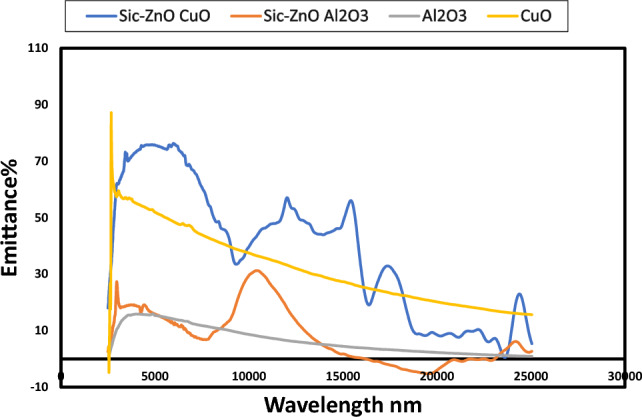
Emittance of deposited SiC and ZnO above Al_2_O_3_ and CuO and two oxides Al_2_O_3_ and CuO.

Figure 6 shows the optical absorbance of CuO and Al_2_O_3_ before and after deposition of ZnO and SiC thin films above them. Absorbance of CuO about 89% and for Al_2_O_3_ about 75% with the same pattern at UV, visible light and IR. Multilayer structure CuO/ZnO/SiC had absorbance about 85% and Al_2_O_3_/ZnO/SiC structure had absorbance about 65%. Wang et al.^7^ deposited CuO/ZnO and reported its optical properties. They found a red-shifted absorption edge at 370 nm, which is not existed in this work. The deposition of SiC thin film above CuO/ZnO structure enhanced its optical properties, moreover absorbance reached 85% as shown in Fig. 6. Figure 7 illustrates emittance of CuO and Al_2_O_3_ before and after deposition of ZnO and SiC thin films above them.

Zno and SiC are semiconductors that show high IR reflectance (low absorbance) and a relative steep edge in the visible region, which is a well-known, metal like behaviour. The relative steep edge refers to inter band transitions involving the d-type free electrons, which means it contains conduction electrons resulting in metal-like electrical conductivity^40,41^. At CuO/ZnO/SiC structure higher incident energies at wavelength (215–730 nm), interband transitions take place, then absorbance decreased gradually. Moreover, the increasing of absorbance is attributed to the light scattering and increased light trapping at wavelength (215–730 nm). As mentioned before, CuO/ZnO/SiC structure has a higher roughness than other one. The scattering light may have occurred due to the surface roughness, whereas roughness is attributed to effect on surface characterization. Surfaces with high degree of roughness that imply the possibility of using the porous layer as an antireflection coating because the surface reduces the light reflection and increase absorbance^42^.

CuO/ZnO/SiC multilayer structure had absorbance higher than Al_2_O_3_/ZnO/SiC, but selectivity (absorbance/emittance (α/ε)) of Al_2_O_3_/ZnO/SiC structure (0.65/0.15) is better than CuO/ZnO/SiC structure (0.85/ 0.5). Spines, spheres, and valleys shown in CuO/ZnO/SiC in AFM images had a good effect on absorbance and increased the emittance, which resulted in a low performance of this structure. However wavy morphology of Al_2_O_3_/ZnO/SiC resulted in good selectivity of this structure.

This work was demonstrated to evaluate two multilayer structures as selective absorbers for thermal solar energy (medium temperatures). Previous works deposited SiC or ZnO thin films alone and evaluate or studied their properties. This work’s target was making a multilayer structure from ZnO and SiC with two different substrates and two different oxides (CuO and Al_2_O_3_) to show the effect of this structure on morphology, structure, and optical properties. ZnO and SiC thin films were chosen due to their high thermal stability, electronic properties, in addition to good optical properties. ZnO became a suitable and an efficient candidate for a material in solar cells third generation, due to many advantages such as good growth control, large energy bandgap, low cost, and high electron mobility. SiC thin films have unique properties, such as high thermal stability, good chemical resistance, good optical properties, and due to its wide band gap, it can be used in new advanced applications to develop efficient UV photonic. Moreover, semiconductors absorb short wavelengths of light due to their band gap and have low emittance. Morphology of each structure was studied, and its effect on the optical properties was discussed. Emittance is related to surface properties such as roughness of surface and the mean height deviations^2^. Table 1 showed that CuO/ZnO/SiC structure had a higher average of surface characteristics than the other structure. Meanwhile, it was reflected on the increasing of absorbance and emittance of CuO/ZnO/ SiC structure.

Figure 2 b,d showed that CuO had a higher thickness (about 5 times) than Al_2_O_3_. Although ZnO and SiC thin films were deposited with the same conditions, they had different morphology and optical properties. The higher thickness of CuO had a direct effect on deposited thin films morphology and optical properties. Saklayen et al. studied the effect of film thickness on the morphology and optical properties^43^. They found that the mean grain area, average (Ra), and roughness are increased with increasing film thickness. These findings are consisted with this work results. CuO had the higher thickness, so CuO/ZnO/SiC structure had the higher roughness and higher crystallite size.

## Conclusion

In this work two multilayer structures were characterized and evaluated as solar selective absorbers for medium temperature thermal solar energy applications. The two investigated multilayer structures are CuO/ZnO/SiC and Al_2_O_3_/ZnO/SiC. Both structures CuO/ZnO/SiC and Al_2_O_3_/ZnO/ SiC presented almost continuous pattern in UV and visible light ranges, but the higher absorbance in UV, visible light and short IR was related to CuO/ZnO/SiC multilayer structure. CuO/ZnO/ SiC exhibited absorbance and selectivity of about 85% and 0.85/0.5, respectively. Al_2_O_3_/ZnO/SiC demonstrated absorbance and selectivity of about 65% and 0.65/0.15, respectively. This work has discussed the effect of surface topography, roughness, max. height profile, and waviness of the surface on optical properties. Increasing the roughness of the surface had a positive effect on absorbance, but it increased the emittance of the surface as well.

## Data Availability

Data will be available upon reasonable request to the corresponding author.

## References

[1] Bello M, Shanmugan S (2020). Achievements in mid and high-temperature selective absorber coatings by physical vapor deposition (PVD) for solar thermal application-a review. J. All. Comp..

[2] Kennedy CE (2002). Review of Mid. to high-temperature solar selective absorber materials. Natl. Renew. Ener. Lab..

[3] Barshilia HC (2014). Growth, characterization and performance evaluation of Ti/AlTiN/AlTiON/AlTiO high temperature spectrally selective coatings for solar thermal power applications. Sol. Energy Mater. Sol. Cells..

[4] Kumari Y, Jangir LK, Kumar A, Awasthi K (2021). ZnO in solar cell and ultraviolet detectors. Metal Oxides. Nanostructured Zinc Oxide.

[5] Wojcik PM, Bastatas LD, Rajabi N, Bakharev PV, McIlroy DN (2021). The effects of sub-bandgap transitions and the defect density of states on the photocurrent response of a single ZnO-coated silica nanospring. Nanotechnology.

[6] Amakali T, Daniel LS, Uahengo V, Dzade NY, De Leeuw NH (2020). Structural and optical properties of ZnO thin films prepared by molecular precursor and sol–gel methods. Crystals.

[7] Wang W (2020). Optical properties of CuO/ZnO composites prepared by mechanical grinding. Optik..

[8] Ismail A, Abdullah MJ (2013). The structural and optical properties of ZnO thin films prepared at different RF sputtering power. J. King Saud Uni. Sci..

[9] Sharmila B, Singha MK, Dwivedi P (2023). Impact of annealing on structural and optical properties of ZnO thin films. Microelectron. J..

[10] Sun K (2022). Synthesis and potential applications of silicon carbide nanomaterials/nanocomposites. Ceram. Int..

[11] Ferhati H, Djeffal F, Bendjerad A, Saidi A, Benhaya A (2021). Post-annealing effects on RF sputtered all-amorphous ZnO/SiC heterostructure for solar-blind highly detective and ultralow dark-noise UV photodetector. J. Non-Cryst. Sol..

[12] Tavsanoglua T, Zayimb EO, Agirsevenc O, Yildirimd S, Yucelc O (2019). Optical, electrical and microstructural properties of SiC thin films deposited by reactive dc magnetron sputtering. Thin Sol. Films..

[13] Gholizadeh M, Zarei Moghadam R, Mohammadi AA, Ehsani MH, Rezagholipour Dizaji H (2020). Design and fabrication of MgF2 single-layer antireflection coating by glancing angle deposition. Mat. Res. Innova.

[14] Zarei Moghadam R, Omrany AH, Taherkhani M, Shokrian F (2021). Fabrication of multi-layer antireflection coating consisting of ZnS and MgF2. Prog. Phys. Appl. Mat..

[15] Ashrafi MMA, Dizaji HR, Ehsani MH, Zarei Moghadam R (2018). ZnS film properties modification using oblique angle deposition technique. Surf. Rev. Lett..

[16] Tang L, Cao F, Li Y, Bao J, Ren Z (2016). High performance mid-temperature selective absorber based on titanium oxides cermet deposited by direct current reactive sputtering of a single titanium target. J. Appl. Phys..

[17] Barshilia HC, Selvakumar N, Vignesh G, Rajam KS, Biswas A (2009). Optical properties and thermal stability of pulsed-sputter-deposited alxoy/al/alxoy multilayer absorber coatings. Sol. Energy Mater. Sol. Cells..

[18] Thomas NH (2017). Semiconductor-based multilayer selective solar absorber for unconcentrated solar thermal energy conversion. Sci. Rep..

[19] Tibaijuka JJ, Nyarige JS, Diale M, Mlyuka NR, Samiji ME (2023). Structural and optical properties of DC sputtered Al_x_O_y_/Cr/Al_x_O_y_ multilayer selective absorber coatings. Phys. B: Conden. Matter..

[20] Dan A (2023). Emissivity measurements of W/TiAlN/TiAlSiN/TiAlSiON/TiAlSiO -based multilayer spectrally selective absorbers at high temperature. Sol. Energy.

[21] Wu Y (2021). Enhanced thermal stability of the metal/dielectric multilayer solar selective absorber by an atomic-layer-deposited Al_2_O_3_ barrier layer. Appl. Sur. Sci..

[22] Kamrul Md, Khan A (1999). Copper oxide coatings for use in a linear solar Fresnel reflective concentrating collector. Renew. Energy.

[23] Abdelfatah A, Mohamed LZ, Elmahallawi I, Abd E-F (2023). Comparison of structure and solar-selective absorbance properties of Al_2_O_3_ thin films with Al and Ni reflector interlayers. Chem. Pap..

[24] Lazarov BM, Sizmann R, Goff AH, Granqvist CG, Lampert CM (1992). Calorimetric measurements of the total hemispherical emittance of selective surfaces at high temperatures. Proc. SPIE.

[25] Marin E, Lanzutti A (2013). Tribological properties of nanometric atomic layer depositions applied on AISI 420 stainless steel. Tribol. Ind..

[26] Mwema FM, Oladijo OP, Sathiaraj TS, Akinlabi ET (2018). Atomic force microscopy analysis of surface topography of pure thin aluminium films. Mater. Res. Express.

[27] Petrov I, Barna PB, Hultman L, Greene JE (2003). Microstructural evolution during film growth. J. Vac. Sci. Technol. A..

[28] Abd El-Fattah H, El Mahallawi I, Shazly M, Khalifa W (2020). Microstructure evolution of NiTi magnetron sputtered thin film on different substrates. Key Eng. Mat..

[29] Ujjain SK, Roy PK, Kumar S, Singha S, Khare K (2016). Uniting superhydrophobic and lubricant infused slippery behavior on copper oxide nano-structured substrates. Sci. Rep..

[30] Aqil M, Azam M, Aziz M, Latif R (2017). Deposition and characterization of molybdenum thin film using direct current magnetron and atomic force microscopy. J. Nano Technol..

[31] Rana SB, Bhardwaj VK, Singh S, Singh A, Kaur N (2012). Influence of surface modification by 2-aminothiophenol on optoelectronics properties of ZnO nanoparticles. J. Exper. Nanosci..

[32] Masud RA (2020). Preparation of novel chitosan/poly (ethylene glycol)/ZnO bionanocomposite for wound healing application: Effect of gentamicin loading. Materialia..

[33] Wang FL, Zhang LY, Zhang YF (2009). SiC nanowires synthesized by rapidly heating a mixture of SiO and Arc-discharge plasma pretreated carbon black. Nanoscale Res. Lett..

[34] Kundu K, Chakraborty J, Kumar S, Prasad NE, Banerjee R (2020). Enhancement of optical properties of boron-doped SiC thin film: A SiC QD effect. Bull. Mater. Sci..

[35] Ansari SA, Husain Q (2011). Immobilization of Kluyveromyces lactis galactosidase on concanavalin A layered aluminium oxide nanoparticles: Its future aspects in biosensor applications. J. Molecul. Catal. B: nzym..

[36] Ashok CH, Rao KV, Shilpa Chakra CH (2014). Structural analysis of CuO nanomaterials prepared by novel microwave assisted method. J. Atoms Mol..

[37] Tamuly C, Saikia I, Hazarika M, Das MR (2014). Reduction of aromatic nitro compounds catalyzed by biogenic CuO nanoparticles. RSC Adv..

[38] Fissel A, Pfennighaus K, Kaiser U, Schröter B, Richter W (1997). Hexagonal and cubic SiC thin films on SiC deposited by solid source MBE. Diamond Rel. Mater..

[39] López-Suárez A, Acosta D, Magaña C, Hernández F (2020). Optical, structural and electrical properties of ZnO thin films doped with Mn. J Mater. Sci.: Mater. Electron..

[40] Vavilov, V.S. Absorption of light by semiconductors. in *Effects of Radiation on Semiconductors*. Springer (1965).

[41] Waki I, Hirai Y (1989). The silicon L-edge photoabsorption spectrum of silicon carbide. J. Phys.: Condens. Matter..

[42] Abood HK, Mutlak FA-H (2020). Structural, morphological and optical properties of n-type porous silicon-effect of etching current density. IOP Conf. Series: Mater. Sci. Eng..

[43] Saklayen G, Islam S, Rahman F, Ismail AB (2014). Investigation on the Effect of film thickness on the surface morphology, electrical and optical properties of e-beam deposited indium tin oxide (ITO) thin film. Adv. Mater. Phys. Chem..

